# Methodical Considerations and Resistance Evaluation against *F. graminearum* and *F. culmorum* Head Blight in Wheat. The Influence of Mixture of Isolates on Aggressiveness and Resistance Expression

**DOI:** 10.3390/microorganisms8071036

**Published:** 2020-07-13

**Authors:** Akos Mesterhazy, Andrea Gyorgy, Monika Varga, Beata Toth

**Affiliations:** 1 Cereal Research Non-Profit Ltd., 6726 Szeged, Hungary; varga.j.monika@gmail.com (M.V.); beata.toth@gabonakutato.hu (B.T.); 2 NAIK Department of Field Crops Research, 6726 Szeged, Hungary; gyorgyandrea88@gmail.com

**Keywords:** disease index (DI), fusarium damaged kernels (FDK), deoxynivalenol (DON), host-pathogen relations, phenotyping FHB

## Abstract

In resistance tests to Fusarium head blight (FHB), the mixing of inocula before inoculation is normal, but no information about the background of mixing was given. Therefore, four experiments (2013–2015) were made with four independent isolates, their all-possible (11) mixtures and a control. Four cultivars with differing FHB resistance were used. Disease index (DI), Fusarium damaged kernels (FDK) and deoxynivalenol (DON) were evaluated. The isolates used were not stable in aggressiveness. Their mixtures did not also give a stable aggressiveness; it depended on the composition of mix. The three traits diverged in their responses. After the mixing, the aggressiveness was always less than that of the most pathogenic component was. However, in most cases it was significantly higher than the arithmetical mean of the participating isolates. A mixture was not better than a single isolate was. The prediction of the aggressiveness level is problematic even if the aggressiveness of the components was tested. Resistance expression is different in the mixing variants and in the three traits tested. Of them, DON is the most sensitive. More reliable resistance and toxin data can be received when instead of one more independent isolates are used. This is important when highly correct data are needed (genetic research or cultivar registration).

## 1. Introduction

Mixing of isolates is a general methodical procedure used to produce inoculum for artificial inoculation. In most cases, no reason is given as to why it is used. It is known that the isolates of the *Fusarium* spp. have a strong variability in aggressiveness [1,2,3]. As mixing in seedling tests strongly influences aggressiveness [1], it is important to know what the influence of mixing on the disease-causing capacity is. It is clear now that *Fusarium graminearum* and *Fusarium culmorum* do not have vertical races and the resistance is race-non-specific [4,5,6,7]. Another important feature is that the resistance is also species-non-specific [8,9], meaning that the same quantitative traits locus (QTL) gives protection against all the *Fusarium* species tested. Highly significant differences were detected in aggressiveness within the *F. graminearum* and *F. culmorum* populations [4,10,11,12]. In addition, the aggressiveness does not seem to be stable [13], as proven by the many significant isolate/year interactions [14,15].

In this paper, and our previous publications, we used the aggressiveness term for the disease-causing capacity of the given inocula, as virulence is taken for the race-specific pathogens like rusts. The term pathogenicity is referred to the disease-causing capacity of the genus itself [4].

Table 1 shows a cross section of the literature working with mixtures. Research task, plant, media for increasing inoculum, conidium concentration, number of participating isolates in the inoculum and the data of visual symptoms, Fusarium damaged kernels (FDK) and deoxynivalenol (DON) were followed.

Numerous authors used spray inoculation (*n* = 22) [18,20,22,24,28,31,39,46,62,63], and point inoculation was used in 20 cases [16,23,40,49,53]. The Chinese authors work mostly with point inoculation [21,26,27,29,37,41,45]. Many American sources also use this [5,25,26], partly with Chinese scientists working in the US, or from US–China collaboration. However, in increasing numbers, spray inoculations. In some cases, papers are found where both inoculation methods are used parallel [16,24,25,37,40]. Mixtures are made mostly from different isolates of the same *Fusarium* species; in several cases, the different chemotypes are mixed. However, without mixing, no tests were made, so nothing can be said about the effect of the mixing. We have an example that the inoculation was made separately with *F. graminearum* and *F. culmorum*, and then data were pooled for ANOVA [24]. The number of isolates in the mix varied from 2 to 39. In the eight cases, the participating inocula were adjusted before mixing to the given concentration, and then in three inocula, one-third of the amount was pooled to secure the same rate of the given inocula in the pooled version. For the others, we do not have such information, and in several cases no isolate number was given; this case is marked with “more” in the column no. of isolates in Table 1. Aggressiveness before the test was made only in one case [40]; for others, no test was performed. In several cases, the selection of the isolates was made based on experience of earlier years. The conidium concentration is very variable from 10,000 to one million. There is no explanation for this. This means that besides the mixing, the adjusting conidium concentration can also cause problems. There are two conclusions. There is no control of aggressiveness from side of the mixing and diluting. Therefore, only after finishing the test will be clear, whether the necessary aggressiveness could have been secured to achieve the necessary reliability of the experiment. The fifty papers were listed, but in four papers, two lines were used as the authors have applied different inoculation methods or different *Fusarium* spp. Thus, the total number of the cases is 54. The aggressiveness level was evaluated by the presented visual data in this paper. Nineteen cases were found in the high to —very high aggressiveness group, 18 were classified medium or medium/high, eight had low or medium/low level, and in ten cases, no data were printed (not tested or not given). From the 54 cases, only 17 proved good and acceptable, the others were of lower level with moderate differentiation power or even less. This shows, clearly, that securing the necessary aggressiveness could be secured at 36% of the cases. In many cases, disease index was found; in other cases, severity was mentioned, but looking at the data average, severity was indicated, so these were also considered as disease index. In older literature, this was normal. FDK severity was tested only in eleven cases; five cases were high, three cases low, and two medium severity. In 37 cases, we have no data. DON was measured in 17 cases, five cases had high numbers, two were medium, and 10 were low or low/medium and medium/low qualifications. It is important that, in several cases, high aggressiveness in visual symptoms resulted in low DON yield in grains [27,34,42,50]. However, in one case, one poor visual rate showed high DON contamination [39]. The data show that the response to visual symptoms, FDK and DON is not the same. The most important task is the reduction of the DON contamination. The problem is that the least research is done in this field, and only in five cases were the data suitable to analyze DON response; this is less than 10% of the cases.

In the cited literature, the number of isolates in the mixtures varied between 2 and 39. The conidium concentration was set to between 5500 and 5 × 10^6^. This leads to the following question: is mixing and adjusting isolates not significant, or does it have a significant influence on inoculation results? From the papers, we did not get any information. The fact that everybody worked with the best thought conidium concentration and mixing—the published results do not support this probable conviction. However, we thought that the questions should be answered. Therefore, one should know what really happens when different isolates are mixed. After the test, we will know more, how the mixing is working and whether the aggressiveness of the composite inoculum could be.

An important thing should also be considered. Suppose that the aggressiveness problem can be solved for the one inoculum used normally (single isolate or mixture); the question remains whether the single inoculum can provide the reliability of the testing needed for scientific purposes in genetic analyses, variety registration trials, etc. Snijders [64,65] applied four *F. culmorum* isolates from Research Institute for Plant Protection IPO-DLO, Wageningen, NL (IPO 39–01, IPO 329–01, IPO 348–01 and IPO 436–01). Ranking of isolates and the height of the infection were different and variety responses showed high variability. Further results also showed significant isolate-year interactions [66,67,68,69,70,71], e.g., changing ranks in different years. Besides the changing isolate ranking, the variety ranking differed, that also can be a problem in resistance classification. It seems [1] that the more aggressive isolates keep their aggressiveness much better (following dilution) than the less aggressive ones. It is supposed that the mixtures may have a similar picture.

Therefore, this study focused on three main objectives. First, making inoculations with four *Fusarium* isolates in every possible combination to observe the range of plant reactions as widely as possible. Second, to gain more reliable information about the response of cultivars with differing resistance levels, and the structure of resistance expression in order to understand the behavior of the isolates and their mixtures, depending on their aggressiveness level, and to study FDK and DON responses. Here, the changing variety ranks are especially important. Third, as the different traits (FHB, FDK and DON) often do not respond the same way, obtaining more information that would promote regulation of these traits at different aggressiveness levels.

## 2. Materials and Methods

### 2.1. Plant Material

Four winter wheat cultivars received from the Cereal Research Nonprofit Ltd., Szeged were tested (Table 2) with differing resistance levels. Their resistance or susceptibility have been verified, years ago, both under natural and artificially inoculated regimes.

### 2.2. Field Conditions and Experimental Design

In the field tests, the recent basic methodologies were followed [70,71]. The tests lasted three seasons (2013, 2014 and 2015). As the mean for FDK was 66% in 2013 and 13.3% 2014, it was decided to continue the experiment. In 2015, two independent tests were performed with the same isolates, but different inocula, so four experiments were performed and evaluated as a unit. The plant material was sown and evaluated in the nursery of the Cereal Research Nonprofit Ltd. in Szeged, Hungary (46°14′24″ N, 20°5′39″ E) (Kecskes Experimental Station). The field experiments were conducted in four replicates in a randomized complete block design. The plot size was 1 × 5 m. For the 16 groups of heads, one plot was planned as a unit. Sowing was done in mid-October by using a Wintersteiger Plotseed TC planter (Wintersteiger GmbH, Ried, Austria). (Temperature data originate from the National Meteorological Station, Szeged, 1000 m from the nursery; precipitation was measured daily at 7.00 a.m. in the Kecskes Station, about 2–3 hundred m from the actual plots.) The weather data were similar in May and June (precipitation 2013 265 mm, 2014 142 mm). Concerning temperature, the monthly means for both years were 17.2 °C in May; in June, 19.9 °C and 20 °C were the corresponding data (2015 also showed very close data). The only difference is that the 2014 January–April had 110 mm rain and 2013 brought 224 mm rain. The driest year was 2015, with 112 mm winter, 68 mm May and 22 mm June precipitation.

### 2.3. Inoculum Production and Inoculation

**Figure 1:**
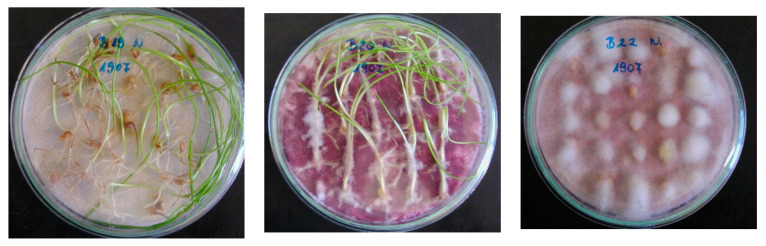
Aggressiveness test with different isolates of a moderately susceptible genotype No. 1907. Low (B19 **left**), medium (B20 **middle**) and highly aggressive (B22 **right**) isolates of *F. graminearum*. Original inocula, without dilution or mixing (the pictures are illustrations to show the aggressiveness differences within *F. graminearum*).

*F. graminearum* and *F. culmorum* are the most important causal agents [72] and two isolates of each species were involved for testing. In the tests, four isolates were used, from *F. culmorum,* the Fc 12375 (1) that were isolated from wheat stalk inside space mycelium from a greenhouse test in the greenhouse of Cereal Research Inst. in 1977. The Fc 52.10 (2) and the two *F. graminearum* isolates, Fg 19.42 (3) and Fg 13.38 (4), originated from naturally contaminated wheat grains (2010). Their monosporic lines were used in the tests. To propagate inoculum the bubble-breeding method was used [1,3,10] on liquid Czapek-Dox medium. As aggressiveness is a variable trait [4,10,69,70], 50% more inocula were produced and the best ones were chosen for use. This way it was possible to put the aggressiveness under control. The aggressiveness of the isolates was done by the Petri dish method [1,3] (Figure 1). The inocula were stored until usage at 4 °C. Since the amount of material from the flowering plots was checked on the previous day, only that amount was separated following careful mixing from content of the 10 L balloon, as was necessary for that given day. The rate of the inocula in the mixtures was 50–50% with two components, one-third for three and one fourth with four components. They were made in the afternoon before inoculation. The suspension was fragmented by the Eta Mira household mixer machine (Czech Republic) with a 1 L volume-mixing unit.

The inoculation was made at full flowering with spray inoculation. First, the control heads were covered at the end of the plot by a polyethylene bag without inoculation, with only sterilized water being sprayed. This was necessary to avoid cross inoculation from the suspension treated groups of heads. Then each plot was inoculated with 15 inocula (isolates 1, 2, 3, 4, 1 + 2, 1 + 3, 1 + 4, 2 + 3, 2 + 4, 3 + 4, 1 + 2+3, 1 + 2+4, 1 + 3+4, 2 + 3+4 and 1 + 2 + 3 + 4) on group of heads within a plot. As the mixtures were mixed *v/v* basis (at three components, one third was given from every component [16,17,18,40,44], in the counting of the effect of the mixing, the arithmetical mean was applied. This was applied earlier [2]. This was proportional with the volumes. When no interaction occurs between components, the arithmetical mean functions. If this is not the case, the arithmetical mean serves as control to compare the performance of the mixtures. In this case, as we had aggressiveness data of the participating components, the effect of the mixing could be measured. Each group of heads consisted of 15–20 heads and about 50 cm distance between each other to prevent cross inoculation between the isolates. The groups were positioned about 30 cm from the edge in two lines, in each, 8 and 7 groups of heads were spray inoculated (about 15 mL suspension each). Following inoculation, the sprayed heads were covered by polyethylene bags for 48 h [69]. As head size was different, larger heads needed more suspension to have the same coverage as smaller ones. After removing the bags, the groups of heads remained loosely bound at half height of the plants, not to disturb assimilation of the leaf system.

### 2.4. Evaluation of the Disease and Toxin Analysis

The evaluation of the visual symptoms was done on 10, 14, 18, 22 and 26 days after inoculation. In warmer years, the 22nd day was the last, because of the yellowing, the head symptoms could not be evaluated properly [4,14]. During evaluation, the percentage of the spikelets in the heads of groups were directly estimated as percentage value. Threshing was made carefully to not lose lighter infected grains (Seed Boy, Wintersteiger AG., Ried, Austria); fine cleaning was performed by an Ets Plaut-Aubry air separator (41290 Conan-Oucques, Conan-Oucques, France). In both cases, air speed was regulated so that light Fusarium infected grain remained. Then a visual evaluation of the FDK grains followed, expressed as a percentage value.

For toxin analyses, 6 g of the individual samples was separated for milling by a Perten Laboratory mill (Laboratory Mill 3310, Perten Instruments, 126 53 Hägersten, Sweden). Toxin extraction and DON toxin analysis was done according to Mesterházy et al. [14], where 1 g of fine milled wheat sample of the 6 g milled grain sample was extracted with 4 mL of acetonitrile/water (84/16, *v/v*) for 2.5 h in a vertical shaker. All chemicals and toxin standards were purchased from Sigma-Aldrich (1117 Budapest, 23. October Street 6–10). After centrifugation (10,000 rpm, 10 min), 2.5 mL of the extract was passed through an activated charcoal/neutral alumina solid phase extraction (SPE) column (Sigma-Aldrich Supelco, volume 5 mL, filled with 1 g mixture of 20 g Al_2_O_3_/Sigma/and 1 g activated carbon coal/Sigma/, prepared in the laboratory) at a flow rate of 1 mL/min. Then, 1.5 mL of the clear extract was transferred to a vial and evaporated to dryness at 40 °C under vacuum. The residue was dissolved in 500 µL of acetonitrile/water (20/80, *v/v*). Liquid chromatographic separation and quantification was made on an Agilent 1260 HPLC system (Agilent Technologies, Santa Clara, CA, USA) equipped with a membrane degasser, a binary pump, a standard autosampler, a thermostated column compartment and a diode array detector. DON was separated on a Zorbax SB-Aq (4.6 × 50 × 3.5 µm) column (Agilent) equipped with a Zorbax SB-Aq guard column (Agilent) (4.6 × 12.5 × 5 µm) thermostated at 40 °C. The mobile phase A was water, while mobile phase B was acetonitrile. Validation of DON was made by using the DON control toxin from Sigma with a regular dilution grade series. So all concentrations were within the scope of the validation line. The gradient elution was performed as follows: 0 min, 5% B; 5 min, 15%B; 8 min, 15%B; 10 min, 5% B; 12 min, 5% B. The flow rate was set to 1 mL/min. The injection volume was 5 µL. DON was monitored at 219 nm.

### 2.5. Statistical Analysis

The visual data for the 4–5 readings for a group of heads were averaged and they served as entries into the ANOVA method. For DON the yield of every group of heads was analyzed for DON. The four-way ANOVA was done according to the functions described in Sváb [73] and Weber [74] with the help of the built-in functions of Microsoft Excel. In the controls for visual evaluation, no visible infection of FHB and FDK was found. The data were not considered in the variance analysis, but the DON showed a low-level contamination in the control, so these data were included in the ANOVA analysis. To evaluate the significance of the two-ways and three-ways interactions, we followed the methodology suggested by Weber [74].

## 3. Results

### 3.1. Visual Data, Disease Index

Table 3 presents the data across experiments. The four isolates had very distinct aggressiveness across experiments (Table 3A). The general tendency is that the most aggressive isolate, Fg 19.42, had the highest value alone, all combinations produced less aggressiveness. On the other hand, the least aggressive isolate showed higher aggressiveness in mixture in all cases than when it was used alone. The two least aggressive isolates gave results closer to the more weakly aggressive Fc 52.10. All eleven mixtures showed lower aggressiveness than the most aggressive component. The difference between inoculum means is highly significant, the maximum is 50.7, the minimum is 5.08, the distance is 45.7, and the limit of significance LSD 5% for the 15 inocula is 2.06 (64 replicated behind each mean). As the mixing was made on *v/v* rate, it was anticipated that without specific mixing effects of the components, the arithmetical mean of the participating components will show the postulated performance. This was not the case. The measured aggressiveness across genotypes was seven cases higher than the mean of the components, and in four cases, the mean was lower than the arithmetical mean. The real data were between 74% and 173%. This means that the resulting aggressiveness is very variable and its aggressiveness cannot be forecasted. At present, we do not know the reason; further research should solve the problem. We can state, however, that the mixing will reduce the aggressiveness of the most aggressive component in each mixed inoculum, but balances much from the lower aggressive components in positive direction. On average, the mean shows a 28% increase in aggressiveness compared to the hypothetic arithmetical mean model. At the same time, the correlation coefficients, all are significant at *p* = 0.001 or higher at r-values between 0.976 and 0.985, indicating a similar response (Table 3B, *n* = 4)). This was not unexpected as we found it many times working with different isolates independently.

As the LSD 5% value 4.12% is valid for any difference among the data in Table 3A, the problem of significance can be identified without problem. When this is smaller than 4.12, no significant difference can be shown, when larger, it is proved. In many cases the behavior of genotypes (lines) or inocula (columns) is not so, and strongly varies. At low aggressiveness, no significant difference in resistance occurs for Isolates Fc52.10 and Isolate 4 Fg13.38 or the mixture 2 + 4. In other cases, such as Fc12375 3 + 4, the differences between genotypes are significant, with nearly 50% difference between them.

The variety reactions were compared for every inoculum (*n* = 15) so that the cultivar data were expressed at each cultivar to the mean of the four cultivars.

When the correlations are counted between the responses of the cultivars to different isolates, from the 105 correlations, only 13 were significant at *p* = 5% (Table 3C). Seven of the 11 mixed inocula contained Isolate 3. For the others, we have (altogether) four cases. The very variable correlations clearly show that the ranking of the genotypes at the different inocula (isolates and their mixtures) present a high diversity. In three inocula, the difference between genotypes is not significant (LSD 5% is smaller than 4.12%. Six of the genotypes have three cases without significant difference. Five cases were with no difference between two genotypes. Only one inoculum presented significant difference between all genotypes. This was the case also for the means of the four cultivars. For us the real problem is here—which inoculum is optimum to present differences in variety resistance? From the disease index, it seems that the mixing did not give to better differentiation of the genotypes. In this respect, the mixing is not the approach that would bring us closer to a more powerful methodology.

The four experiments (Table 4) had the same means for isolates and their combinations, but the means of the experiment differences are much larger, i.e., 44%, 8%, 26% and 28.5% (2013, 2014, 2015a, 2015b). The data proved that the differentiation between genotypes at low infection pressure is rather poor and not reliable compared to the data of the other years (Table 4B). All correlations (*n* = 4) where 2014 is a partner, gave correlations of r = 0.50, r = 0.66 and r = 0.69. For the rest, the correlations are between r = 0.85 and r = 0.99. Looking at the genotype correlations for the four years (*n* = 15) (Table 4C) from the 105 correlations, 52 were significant. Isolates 3 and 4 showed the least significant correlations with other inocula. It seems that aggressiveness level has a much higher importance in experimentation then mixing has. This is partly ecology-dependent, but is a result, also, of interaction between the aggressiveness level and differentiation of genotypes.

### 3.2. Fusarium Damaged Kernels (FDK)

The values of the FDK data (Table 5) are much higher than the DI data; the mean was for DI 26.7% and for FDK 44.9. In the controls, no visual infection was recorded, so all infections originated from the artificial inoculation (Table 5A). The reduction of the aggressiveness through mixing is significant, but in extent, less than that of the FHB values. Here, the difference between the measured FDK and the counted is larger, 36% mean increase could be registered. The combinations having Isolate 3 (Fg 19.42) have a mean higher than 50% and in one case, higher than 60%. The mixing produced data compared to the arithmetical mean of the aggressiveness of the components between 91 and 184%. Actually, every mixture variant has more or less differing aggressiveness levels. The different compositions mean characteristic aggressiveness differences, which also influence the expression of resistance. The correlations between genotype means across years (Table 5B, *n* = 15) highly significant correlation above r = 0.90, indicating the similarity of the response of cultivars to different isolates. However, when we compare the aggressiveness of the mixture and the mean of participating isolates, we receive large deviations. From 91% to 184%, every possibility can occur. In Table 5C (*n* = 4) where the isolate reactions were compared for genotypes (*n* = 4), the variability in the correlations grow significantly, indicating different responses of the genotypes to the individual inocula.

However, the cultivar responses often differ in the different isolates and mixture. The two more resistant genotypes are the same we found for FHB, GK Fény and GK Csillag. In eight cases, the response of the two cultivars does not significantly differ from each other. In five cases, GK Csillag has lower value, and only in one case GK Fény. GK Garaboly and GK Futár do not show significant difference in six cases; in all other cases, GK Futár has lower values. The larger differences are more stable, GK Csillag has better resistance in each inocula compared to GK Garaboly, but compared to GK Futár, in seven cases, no significant difference was found. From Table 5A, the correlation between the genotype reactions were also computed (Table 5C). Of the 105 possible correlations, 29 were significant. The non-significant correlations varied strongly. This was 29, more than double than was found in DI (13, Table 3C). Of the 29, twenty-five were found between inocula containing Isolate 3 in one or more partner inocula for the correlation test. This shows that FDK provides a closer correlation matrix. This would mean that a mixture automatically does not solve the problem and does not secure a stable level. The problem is as it was for the DI—that in a regular case we have only one test result, and not 15 as in this test. It is sure that a mixture does not provide the increased security of testing we hope from it. Except for the several low aggressive versions we know better to avoid, even the same aggressiveness does not always guarantee the same variety response. Comparing 2 + 3 + 4 and 1 + 2 + 3 + 4 at 59.98 and 58.75 mean aggressiveness in this trait, in the first case, Futár is significantly more susceptible, and in the other case, they have the same number. This means that we have to look for another solution.

The response of the experiment means (Table 6) strongly differs; 2013 gave the highest FDK values, 2014 was five times less and 2015a and 2015b showed similar results to 2013. The response of the different mixtures was very variable. At high infection pressure, there were rather small differences between FDK values at different isolates and mixtures, except isolate 2 and the combination of isolates 2 + 4 that gave significantly less FDK than the others. There is another feature that needs attention. In 2013, isolate 3 gave 84% FDK, in 2014 39.88%. However, at the same performance in 2013 the 2 + 3 gave only 6%, 3 + 4 gave 9.94% and 1 + 3 + 4 only 6.38%. Another example is 1 + 2 + 4 where 72.40% and 0.84% are the two corresponding data. However, in a less epidemic year, independent of the causing agents, the forecasting of the numbers for a heavy epidemic situation is hardly possible.

It seems that the aggressiveness of the mixtures cannot be predicted based on the individual aggressiveness of the four basic isolates (Table 6B, *n* = 15). It depends also on the ecology, but the individual isolates behave differently in most of the mixtures. The correlations data support this, although the 2014 data do so only moderately. Significantly, the data of the higher epidemic situations correlate much better, above r = 0.90, when 2013 and the two 2015 experiments are compared. This agrees well with what we found for FHB data. The correlations between experiment reactions for every inocula were presented (Table 6C, *n* = 4). From the 105 possible correlations, 57 were significant compared to DI in Table 4 with 52 significant correlations. We have non-significant correlations where aggressiveness was very low, such as isolate 2 (Fc 52.10), where no other inocula gave significant correlation with this set.

### 3.3. DON Contamination

The variety specific data (Table 7) show a similar picture to the FDK data. The four isolates showed larger aggressiveness differences, and their combinations showed rather variable performance, depending on their combinations (Table 7A). It seems the mixing resulted in both a decreasing of the aggressiveness level of the most aggressive Fg 19.42 isolate and in a reduction of DON contamination; and this tendency was true not only for DON but also for DI and FDK. On the other hand, it is also true that the aggressiveness of the mixture was, in most cases, significantly higher than the arithmetical means of the mixtures measured by the performance of the DON contamination original of the participating inocula. The isolates and isolate combinations of the low pathogenic isolates showed normally low aggressiveness and low DON levels, indicating that the use of such isolates does not give suitable inoculum for inoculation. The correlations between cultivars against the 15 different inocula (Table 7B, *n* = 15) to the used inocula (individual and mixed), as well as mixed inocula, were highly significant between r = 0.81–0.95. Here, also, the correlations between the means across cultivars and individual performances were given. These were higher, between r = 0.90 and 0.98 than that of the numbers between cultivars. The conclusion is that the means are better resistance indicators than any of the isolates and their mixtures present.

The resistance expression differs in the 15 inocula. The genotypes that did not differ significantly to a given inoculum having lower differences than the LSD 5% is (Table 7A). In twelve cases, GK Garaboly is significantly more susceptible than GK Csillag and GK Fény. The more resistant GK Futár has higher DON contamination to GK Garaboly only in one case. GK Csillag shows lower DON contamination in seven cases than GK Fény, and in five cases, the GK Fény produces less DON. The difference for mean does not reach the significance. This is all independent from the inoculum, should it be pure isolate or mixture. It can be stated, also, that the large resistance differences have the highest chances to be significant, except in the three cases without significant deviation. At lower resistance differences even otherwise significant differences cannot be demonstrated as the data show at different inocula compared with the man cultivar reaction (Table 7A)

**Figure 2:**
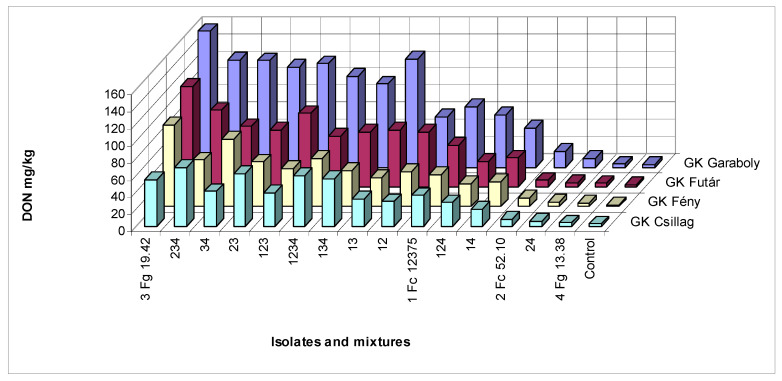
Influence of mixing isolates on the resistance expression of the four cultivars with differing FHB resistance. DON contamination across years and experiments. LSD 5% between any bars in the figure is 18.71.

At two inocula, the cultivars did not show any significant difference. This is true, also, for the control that had natural infection. The DON contamination of the naturally infected heads was low compared to the artificially inoculated samples. On the other side, they are not without risk, as they were higher than the EU limit of 1.25 mg/kg. Four inocula showed three not differing genotypes. Six inocula could not differentiate between two genotypes, and in two cases, every genotype differed from the other. This was the case also for the means. Counting the correlations (Table 7C), being significant, 30 cases were found of the 105 total correlations between inocula. The control values correlated with inocula only in one case. In 24 cases, the Isolate 3 and its combinations showed significant correlations, for the other possibilities we had only six cases. The question is, again, what is the best inoculum? It seems that mixed inocula are not better than the single spore lines are. We have chances for good or very good differentiation at the highest aggressive inocula, but not in every case. For demonstrating no significant differences, we have a chance at low aggressiveness. One solid conclusion is clear. There is no proof that mixtures would be better than pure isolates. However, we should have more inocula than one to present more useful results for a QTL phenotyping or a registration trial. Since, for us, the DON reaction is the most important, we provide the data for it in Figure 2.

There are large differences between experiments (Table 8). The epidemic conditions in 2015 significantly increased the DON contamination; even the FHB and FDK values did not predict these high DON data values (Table 8A). The correlations with the 2014 data and other experiments were moderate, whereas, the others were highly significant with a correlation of r = 0.90 and above (Table 8B). The data also prove that a low aggressiveness level does not allow a proper distinction between the effects of different inocula. As one year does not give well-balanced results, in spite of the higher number of isolates, the tests in different years have a high importance. The correlations between the four experiments were tested (Table 8C). Moreover, 89 cases from the 120 significant relations were registered. This shows a rather good agreement between responses of different experiments. It should be noted that the control values correlated at a high ratio with the artificial inoculation data. For DI 52 and FDK 57, significant correlations were found. It seems that the best correlation matrix was found for DON.

The variance analyses (Table 9) revealed large similarities between the different traits (Table 9A). The three-way interactions were not significant, so an additional F test to the “Within” category was not necessary. All main effects were highly significant. It is more important that the interactions were much smaller than the individual main effects were (Table 9B). This is significant in all cases in differences between the main effect and interactions for FHB and FDK. The variety effect against A × B is not significant for DON indicating that the main effect is more impressionable than the FHB or FDK are. The genotype (A) effect is significant over A × C interaction, indicating a good stability of resistance.

### 3.4. Interrelations between Traits

**Figure 3:**
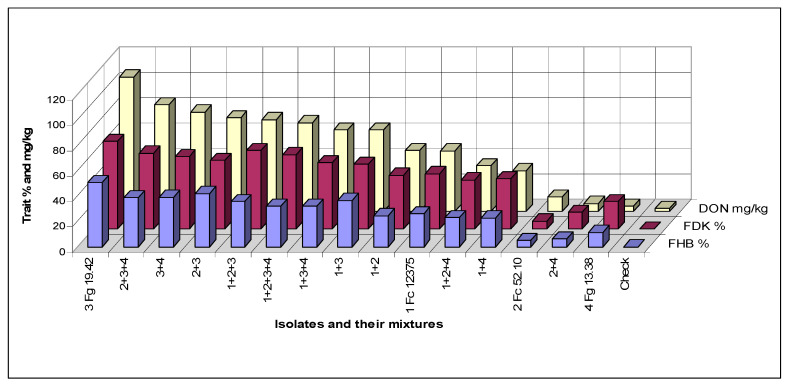
Mixtures and aggressiveness, means for the three traits, Szeged, 2013–2015. LSD 5% between any data for DI 2.06%, FDK 3.82% and DON 9.35 mg/kg.

**Figure 4:**
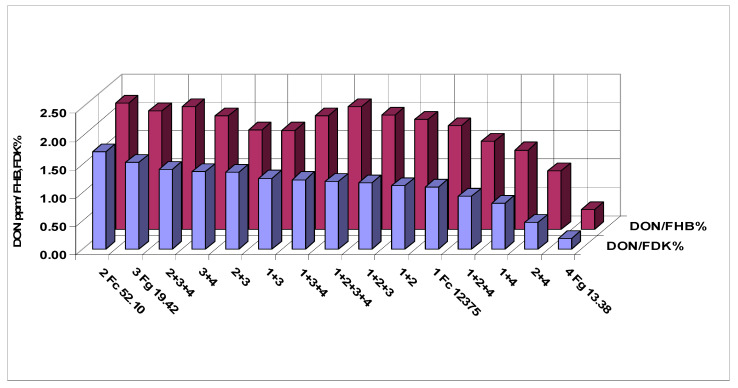
Influence of mixtures on FHB test results. DON production for one percent FHB and FDK infection, 2013–2015.

Figure 3 shows the means of the three traits of the different tested inocula. The data correlate well, but we see that isolates 2 and 4 behave differently from the others. Isolate 4 causes relatively more FHB and FDK, but it is poor in DON production. Isolate 2 behaves oppositely, here the visual data are much lower, but the DON data are higher. Their mixture shows well the transitional result between components. The correlations are very close, they are between r = 0.95 and 0.97 (calculated without the control). The rates for DI/DON and FDK/DON were also calculated (Figure 4). For 1% FDK we had DON content between 1.53 to 0.19 mg/kg. Isolate 3 and its mixtures gave values between 1.25 and 1.53, the others between 0.19–1.12. This clearly means that the forecast of DON contamination via FDK is not possible. We draw also similar conclusions for the DON/FHB rate; however, the different behavior of the DI/DON rate is clear, following a decrease to 1 + 3 the rate increases again. It shows also that the FDK is more precise to describe the DON relation than DI is.

**Figure 5:**
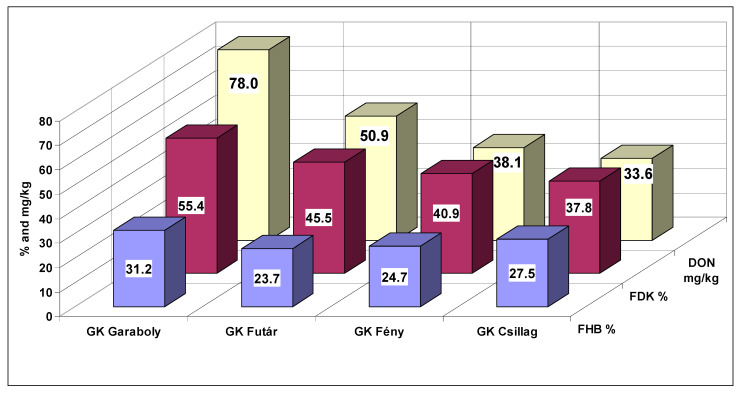
General performance of the cultivars across experiments and inocula for FHB (DI), FDK and DON contamination. LSD 5%: DI: 1.06, FDK: 1.97, DON: 4.68.

The cultivar reactions as means across inocula and years illustrate the well-known fact that visual head data are less informative for DON than the FDK data are (Figure 5). Based on the FHB values, no DON forecast is possible. GK Csillag is important as this cultivar performed well after a fungicide treatment than any other cultivars tested. Based on data from the milling industry, all staples were bought from this cultivar in 2010 when we had the national FHB epidemic. The correlation between FHB and DON is r = 0.66, which is not highly significant, whereas between FDK and DON the correlation is r = 0.9968, and is significant at *p* = 0.001. It means that the FDK signalizes the DON content much better than the visual scores.

**Figure 6:**
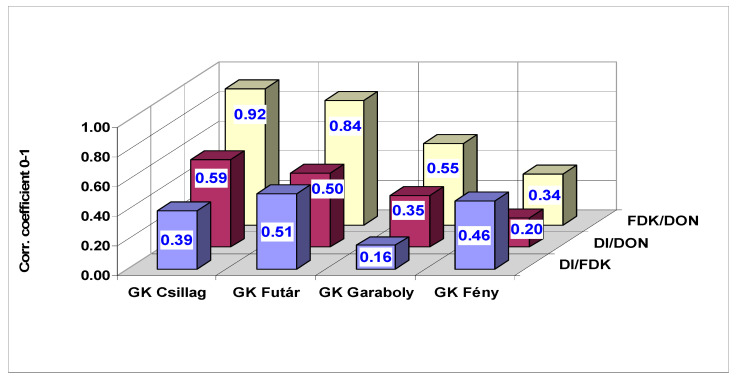
Correlations between genotype reactions to different FHB traits (data from Figure 2, Figure 3 and Figure 4, for each isolate the performance of the cultivars was related to the mean of genotypes). Limit for *p* = 0.05 = 0.51, *p* = 0.02 = 0.59, *p* = 0.001 = 0.77.

Correlations were counted between DI, FDK and DON for every cultivars tested to see whether there are different variety to the 15 inocula responses or not (Figure 6). The first conclusion is that the FDK/DON correlations are much closer than the DI/DON correlations, this agrees well with earlier experience. The DI/FDK data did not show any significant relations in the four cultivars, indicating that the same cultivar might have different responses at different inocula and mixtures of inocula. The correlations between DI, FDK and DON were counted also for all isolates separately for the four cultivars (Table 10). In five inocula, all three correlations are highly significant and nearly no difference exist between them. Two of them were single isolates and three mixtures were found (1 + 3, 1 + 4 and 1 + 2 + 4). In eight cases, the FDK/DON correlations were closer than the DI/DON correlations. Thus, it seems that the isolates and their mixtures may have different profiles that can influence resistance expression. The only advantage of the mixture is that the very diverse behavior of 2 Fc 52.10 and 4 Fg 13.38 could be balanced by the mixing to some extent by increasing the aggressiveness. It is important that the DI/DON correlation across all inocula is much lower (r = 0.6668) than the FDK/DON relation (r = 0.9968). The DI/FDK is also low (r = 0.6170). This means that for forecasting DON the FDK is more precise means than visual scores are. This is an argument that FDK and DON contaminations should be treated more seriously than it normally happens.

## 4. Discussion

### 4.1. Influence of Mixing

The mixing of isolates is a generally applied method. This is done, not only to regulate aggressiveness, but it can be a necessity when a larger amount of inoculum is needed than the present methods can produce. The cited literature (16–63) is not consequent. The number of components strongly varies. Moreover, many authors seemingly feel that they are more secure when more isolates are mixed, and they think that a mixture comes closer to the variability found in nature. However, we did not find anything pertaining to this problem in the literature. Thus, it was not clear what the result of the mixing considering the aggressiveness is. As nobody, except one [40], used the aggressiveness test, the mixing was made without an aggressiveness control. The lesson is that the different mixtures did not provide the same aggressiveness. For this reason, nobody can hope that a mixture automatically will have high aggressiveness. This was not an accident; the experimental results do not support such assumptions. Additionally, the reactions differed between DI, FDK and DON contamination. Large and significant differences were found between the different mixtures and pure isolates. There is another problem; that the conidium concentration was adjusted to different values. Of course, this could influence the effect of mixing. Most people used 5 × 10^4^ or 5 × 10^5^ conidia/mL, but (once) 10^6^ conidia/mL was applied. Here, we remark only that to the uncontrolled mixing and uncontrolled adjusting of conidium concentration was a general rule. The effect of dilution was influenced by the isolate, the host variety through resistance, and the aggressiveness level, but other agents may also be present. In laboratory and greenhouse seedling tests [1], mixing of isolates did not give the mean of aggressiveness of the participating inocula aggressiveness in greenhouse experiments and the different mixtures reacted differently. However, these greenhouse tests could not provide any information about the behavior of the mixtures for field conditions. In the best case, it was mentioned in the papers that the isolates were aggressive in earlier tests and, therefore, the authors hoped in a successful infection. Therefore, the general experimental praxis and the earlier data pressed us to investigate this problem.

The result of the mixing depends most on the aggressiveness level of the participating inocula. From the low pathogenic isolate, no highly aggressive inocula can be produced, but from an aggressive isolate from the same test tube, inocula with differing aggressiveness could be produced [75]. When we see the most aggressive isolate (inoculum), the mixture decreases its aggressiveness (each case compared to the most aggressive component). When we compare the aggressiveness of the mixture to the arithmetical mean of the individual components, it becomes clear that in most cases the aggressiveness is higher, with even 50–100% difference in some cases than the mean aggressiveness level counted for the participating inocula. On the other side, the resulting aggressiveness was always lower than that of the most aggressive component. Therefore, the exact forecasting of the result of the mixing is hardly possible. This means that the most aggressive isolate can significantly overbalance the arithmetical mean, but still does not reach the level of the original aggressiveness of the most aggressive component. The system is much more complicated than it was thought earlier. The DI/DON and FDK/DON rates showed an interesting picture. The DI/DON rate was much more variable; this was found mostly when Fc. 12,375 isolate was a participant of the mixture or was alone. We do not know whether this, an instinct attribute, is for other variants of the DI/DON and FDK/DON rations, which were rather similar. It is also considerable that Fg 13.38 has a relatively high DON and FDK ratio and a very low DON content, the other three isolates perform similarly with lower DI, medium FDK and higher DON contamination with strong differences. This is supported also by the higher number of significant correlations from Table 3C, Table 5C and Table 7C.

Uncontrolled mixing and diluting may result in low aggressiveness level and bad differentiation of the genotypes. The only advantage is that it stabilizes the variation in aggressiveness of the inocula compared to the individual components. However, it does not solve the problem of the use of one inoculum. As we do not have races at hand, the most important argument (the mixtures represent a better scope of the pathogenic population) is refuted. In this test series, this view could not be supported. The question is which of the 15 the best solution is. We cannot say each inoculum behaved differently. Having an inoculum the resistance can be underestimated (high aggressiveness) or underestimated low aggressiveness; therefore, several aggressiveness levels are needed to reach more reliable results. On the other hand, the different differentiation of the genotypes at different aggressiveness levels needs a correction mechanism, by the use of more isolates to produce more reliable results. This has a significance in QTL analysis where the large differences can be evaluated well, but the chance to identify for lower or medium effective QTL is not very convincing. The conclusion is that the mixing does not solve the problem to produce top quality inoculum for artificial inoculation. Even the aggressiveness of the participating inocula is known, an exact forecast of the aggressiveness of the mixture is not possible—especially when we would like to know FDK and DON reaction. In resistance response, the mixtures are not better, or worse, than a single aggressive inoculum.

There is another problem. As in this test, also, the case is, the mean performance of different independently used inocula produce a mean that describes much better the resistance level than any of the inocula alone. Earlier research [69,70,71] supports its usefulness. The use of more independent isolates is costly. Therefore, it should be used where high quality phenotyping is necessary for genetic research and other scientific purposes. It is needed to qualify new cultivars with higher or high resistance to FHB. The risk analysis needs careful work. The behavior of the most aggressive isolate resembles well to the combining ability of a given crossing partner in different crosses. In plant breeding, this is a well inheritable trait. It is supposed that such research would be important to make here, to be able to select the isolates that give the highest aggressiveness if possible.

An aggressiveness test is necessary to qualify inoculum before use. No mixing, no adjusting inoculum can secure the high aggressiveness needed for efficient work. When we want to have a mixture, an aggressiveness test can help choose the suitable inocula for mixing.

### 4.2. Resistance Expression

The correlations between cultivar reactions to the 15 isolates and mixtures for DI are between r = 0.96 and 0.98, *p* = 0.001. However, we have about tenfold difference in aggressiveness. From the 15 aggressiveness level the different inocula and their mixtures represent, only 1–2 cases were found where all genotypes significantly differed from each other. Normally two or three genotypes should have been marked without significant difference for the given inoculum. The data are clear that the DON contamination show closer relation with FDK than with DI. Seeing the three traits, the least reliable difference between genotypes is at disease index; larger is at FDK and the largest is at DON. However, this depends also on ecological conditions. It occurred that a higher DI was followed a poorer FDK and DON presentation. As normally only one data set is given, which is the set of the 15 possible cases shown, is a question. It seems to be clear that one inoculum surely will not describe medium or lower resistance differences, with more isolates, it can be done much better, but large resistance differences can be identified with an aggressive inoculum with a high probability. This is a confirmation of earlier results where resistance component kernel resistance and specific DON resistance were measured [2,4,14,15,70]. Resistance expression depends on the composition of the mixture, resistance level, conidium concentration and ecological conditions, so a prediction of the result of the mixture is hardly possible.

### 4.3. Genetic Aspects

There is a new development in the genetic mapping work. QTLs were identified with different role [76]. QTLs can regulate only visual symptoms, visual symptoms and FDK, FDK and DON and QTLs were identified influencing all three traits. Similar results were published by recent authors [77,78,79,80,81]. This will have the consequence that mapping should be extended to FDK and DON that is generally not done. For the crossing partners, this information is inevitable. From the generally used DI mapping [17,19,21,22,23,24,25,29,30,32,33,37,56,61], this aspect falls out and has only reduced significance of the QTL identified.

### 4.4. Breeding Aspects

Large-scale mass selection can be done well by using a mixture with high aggressiveness. In this case, an efficient negative selection should only be done. This should also be measured otherwise, so much aggressiveness can be lost that the necessary high selection pressure cannot be provided.

In an advanced stage of breeding, it is necessary to determine the amount of resistance in the given variety. As responses to DI, FDK and DON may differ, we should test them with more isolates (inocula) with different levels of aggressiveness. This provides a better answer in phenotyping for genetic studies, cultivar registration tests or any other tasks that need high preciosity.

It is not thought that one should be anxious about high aggressiveness to diminish resistance differences. The results prove the contrary; the resistance differences are much higher at high aggressiveness. The papers using high on very high aggressiveness prove this clearly [17,19,22,27,42,58]. Therefore, to identify DON overproduction or DON resistance at least medium or high aggressiveness is needed [69]. A test [15] with four isolates against FHB on 26 genotypes with variable aggressiveness showed that highly resistant lines and cultivars like Sumai 3 and the Szeged line Sgv/NB/MM/Sum3 yielded 0.9 mg/kg DON, the most susceptible line gave between 110 and 433 mg/kg. It seems that in spite of the high aggressiveness the differentiation of the genotypes remained good, even much better than at use of lower aggressiveness. When flowering differences are 2–3 weeks between cultivars, because of the changing environmental conditions their correct comparison is often problematic. By using control genotypes with known resistance levels, the management of the resistance tests can be done. However, in a genetic analysis, with the same flowering time differences we have problems, because we should treat the whole population as a unit and therefore data are often not comparable and the QTLs are often artifacts. There are also good examples. In the framework of the USWBSI (United States Wheat and Barley Scab Initiative (www:scabusa.org), multilocation ring tests are made every year to test wheat and Barley genotypes where beside DI, also FDK and DON response, is evaluated [82]. We follow this working way since more than 30 years. This is the only way that can secure the lower DON contamination of the next variety generations. We hope only that scientific research will recognize the need.

## 5. Conclusions

The Fusarium resistance testing is a very sensitive experimental system. The pathologically uncontrolled mixing and conidium concentration adjusting cannot secure the stable high aggressiveness needed to receive high differentiation of the genotypes [16,17,18,19,20,21,22,23,24,25,26,27,28,29,30,31,32,33,34,35,36,37,38,39,40,41,42,43,44,45,46,47,48,49,50,51,52,53,54,55,56,57,58,59,60,61,62,63]. Mixing and conidium concentration adjusting, which are applied without controlling by plant pathological methods, cannot secure the stable high aggressiveness needed to receive a correct data on differentiation of the genotypes. It also became clear that FDK or DON specific behavior could not be verified exactly by single inocula. In the mixing variants with the same level of DI, very diverse results for FDK and DON can be recorded. It is also sure that one isolate, or mixture, should it be selected as good as possible, provides only one pathogenic level. Without controlling for aggressiveness of the dilution, concentration or mixing can lead to artifacts. Again, without using more isolates independently, will not have the chance to present the phenotypic data that are necessary to receive reliable data for the identification and validation in medium and low effects of QTL. There are strong arguments to change the resistance testing methodology followed until now.

## Figures and Tables

**Table 1:** Experimental data of Fusarium resistance and pathogenicity tests from papers using mixtures of isolates.

Author	Ref. No.	Plant	Application	Medium	Inoculation S or P	*Fusarium* spp.	No. of Isolates	Con. Conc.	Aggressiveness Visual	FHB Visual	Vis. Min.–Max.%	FDK	FDK Min.–Max.%	DON	DON Min.–Max. mg/kg
Andersen et al. 2015	[16]	wheat	Path	MBA	S	gram.	10 *v/v*	5 × 10^4^	medium	DI *	20–50	no	no	high	7–75
Andersen et al. 2015	[16]	wheat	Path	MBA	P	gram.	10 *v/v*	5 × 10^4^	medium/low	DI *	30–40	no	no	low/med	5–15
Garvin et al. 2009	[17]	wheat	QTL	n.g.	P	gram.	3 *v/v*	1 × 10^5^	very high	DI	20–100	no	no	no	no
Busko et al.	[18]	wheat	Path	wheat seed	S	culm	3 *v/v*	5 × 10^5^	no	no	no	no	no	no	no
Pirseyedi et al. 2019	[19]	durum	QTL	n.g.	P	gram.	3	5 × 10^5^	very high	DI	0–100	no	no	no	no
Amarasighe 2010	[20]	wheat	Fung	CMC	S	gram.	7	5 × 10^4^	medium	DI	0–28	medium	7.4–50	Medium	6.8–30.1
Bai and Sharen 1996	[5]	wheat	Res	MBM	P	gram.	n.g.	4 × 10^4^	high	I	7–100	no	no	no	no
Bai et al. 1999	[21]	wheat	QTL	MBM	P	gram	10	4 × 10^4^	low	DI	0.8–10.7	no	no	no	no
Basnet et al. 2012	[22]	wheat	QTL	n.g.	S	gram	n.g.	8 × 10^4^	high	DI	1–90	high	30–90	no	no
Buerstmayr 2002	[23]	wheat	QTL	MBM	P	gram. + culm.	2	5 × 10^4^	high	I	1–9	no	no	no	no
Buerstmayr 2011	[24]	wheat	QTL	MBM	S	gram.	1	2.5 × 10^4^	medium	S (AUDPC)	114–946	no	no	no	no
Buerstmayr 2011	[24]	wheat	QTL	MBM	S	culm.	1	5 × 10^4^	medium	S (AUDPC)	32–967	no	no	no	no
Dong et al. 2018	[25]	wheat	Gen	n.g.	P	gram.	30–39	8–10 × 10^4^	high	I	50–100	no	no	medium	0–30
Dong et al. 2018	[25]	wheat	Gen	n.g.	S	gram.	30–39	8–10 × 10^4^	high	S	20–80	no	no		
Chen et al. 2007	[26]	wheat	QTL	PDA	P	gram.	3	5 × 10^5^	medium	DI	10–55	no	no	no	no
Chu et al. 2011	[27]	durum	QTL	n.g.	P	gram	3	5 × 10^4^	very high	DI	14–75	high	1–100	low	0–38
Cowger et al. 2010.	[28]	wheat	Path	MBB	S	gram.	4 *v/v*	1 × 10^4^, 1 × 10^5^	no	no	no	low	3–18	low/medium	2–16.7
Li et al. 2011	[29]	wheat	QTL	MBM	P	gram.	10	1 × 10^4^	low	DI	0–1	no	no	no	no
Cutberth et al. 2006.	[30]	wheat	QTL	n.g.	P	gram.	3	5 × 10^5^	high	DI	0–100	no	no	no	no
D’Angelo et al. 2014	[31]	wheat	Fung	CMC	S	gram.	10	n.g.	low	DI	1.6–10.4	no	no	very low	0.3–8
Kollers et al. 2013	[32]	wheat	QTL	n.g.	S	gram.	nFg, nFc	5 × 10^4^	medium	DI	0–34	no	no	no	no
Yang et al. 2005	[33]	wheat	QTL	PDA + CMC	S	gram.	4	5 × 10^5^	high	DI	7–97.8	high	2.8–95.7	no	no
Gaertner et al. 2008	[34]	wheat	Res	oat grain	S	culm.	20	5 × 10^6^	high	1–9	2–16	no	no	low/medium	2–16
Evans et al. 2012	[35]	barley	Path	MBA	S	gram.	3	2 × 10^5^	n.a.					high	2–4/spike
Perlikowsi et al. 2017	[36]	trit.	Gen	n.g.	S	culm.	3	5 × 10^4^	medium	DI	12–24	high	14–82	high	0.5–113
Yi et al. 2018	[37]	wheat	QTL	n.g.	P	gram.	4	1 × 10^5^	low	DI	0.58–96	no	no	no	no
Yi et al. 2018	[37]	wheat	QTL		S	gram.	4	1 × 10^5^	medium	DI	0.9–24.4	no	no	no	no
Gervais et al. 2003	[38]	wheat	QTL	barley seed	S	culm.	more	1 × 10^6^	medium	1–9	2.5–8	no	no	no	no
Goral et al. 2002	[39]	trit.	Res	n.g.	S	culm.	10	1 × 10^5^	low	S	7–20	no	no	high	7.5–118
Goral et al. 2015	[40]	wheat	Gen	n.g.	S	culm.	3 *v/v*	5 × 10^5^	medium	DI	8–33	medium	15–37	low	2.5–7.6
Goral et al. 2015	[40]	wheat	Gen	n.g.	P	culm.	3 *v/v*	5 × 10^5^	medium	DI	4.8–32	no	no	no	no
Hao et al. 2012	[41]	wheat	Gen	n.g.	P	gram.	more	5 × 10^4^	n.a.	DI	n.g.				
He et al. 2013	[42]	wheat	Res	LBA	S	gram.	more	5 × 10^5^	high	DI	0–89	no	no	low/med.	0.1–21.4
He et al. 2014.	[43]	wheat	Res	LBA	S	gram.	5	5 × 10^5^	low	DI	0–15.9	low	5–42	low	0.2–7.05
Hilton et al. 1999	[44]	wheat	Res	PDA	S (?)	4 F. spp.	4 *v/v*	2.5 × 10^5^	high	DI	20–78	no	no	no	no
Chen et al. 2005	[45]	wheat	Gen	n.g.	P	gram.	4	n.g.	n.g.	DI	S-MR	no	no	no	no
Klahr et al. 2007	[46]	wheat	Res	n.g.	S	culm.	more	1 × 10^6^	medium/high	DI(AUDPC)	65–1403	no	no	no	no
Lin et al. 2006	[47]	wheat	QTL	n.g.	S	gram.	4	n.g.	low	I	0.05–0.82	no	no	no	no
Liu et al. 1997	[48]	wheat	Res	PDA	S	culm.	8	1 × 10^5^	medium	DI	10–85	high	33–70	med./low	4–16
Forte et al. 2014	[49]	wheat	Gen	n.g.	P	gram.	more	5 × 10^4^	high	DI	8–99	no	no	no	no
Malihipour et al. 2015	[50]	wheat	Gen	n.g.	S (field)	gram.	4	5 × 10^4^	high	I, S	2–87	low	1–23	low	0.2–4.2
McCartney et al. 2015	[51]	wheat	QTL	n.g.	S	gram.	5	5 × 10^4^	medium	DI	15–55	no	no	no	no
Muhovski et al. 2012	[52]	wheat	Gen	n.g.	P	gram.	more	1 × 10^5^	n.g.	DI	n.g.	no	no	no	no
Osman et al. 2015	[53]	wheat	Res	rice grain	P	gram.	2 and 5	1 × 10^5^	n.a.	DI	ng	no	no	no	no
Jones et al. 2018	[54]	wheat	Fung	oat grain	S	mix 5 F. spp.	20	1 × 10^5^	medium	DI(AUDPC)	653	no	no	low	0–3.13
Liu et al. 2019	[55]	wheat	QTL	spawn	n.a.	gram.	20	n.a.	high	DI	6–83	no	no	no	no
Miedaner et al. 2017	[56]	wheat	QTL	n.g.	P	gram.	3	1 × 10^5^	high	DI	20–100	no	no	no	no
Oliver et al. 2006	[57]	wheat	Gen	n.g.	P	gram.	3	1 × 10^4^	medium/high	DI	5–57	no	no	no	no
Tamburic et al. 2017	[58]	wheat	QTL	Bilay	S	gram.	4	5.5 × 10^3^	high	DI	1.6–67	no	no	high	0.5–47.2
Otto et al. 2002	[59]	durum	QTL	n.g.	P	gram.	3	n.g.	high	DI	15–62	no	no	no	no
Ding et al. 2011	[60]	wheat	Gen	n.g.	S	gram.	4	4 × 10^4^	medium	DI	17–51	no	no	no	no
Klahr et al. 2011	[61]	wheat	QTL	n.g.	S	culm.	more	n.g.	n.g.	n.g.	n.g.	no	no	no	no
Zwart et al. 2008	[62]	wheat	Res	n.g.	S	Fg + Fc	2	1 × 10^5^	medium	I,S,	DI	high	33–68	no	no
Oliver et al. 2006	[57]	wheat	QTL	n.g.	P	gram	3	n.g.	n.g.	DI	7.1–57.5	no	no	no	no
Miedaner et al. 2006	[63]	wheat	QTL	n.g.	S	culm.	2	5 × 10^5^	medium	DI	6–35	no	no	high	3–60

The whole table: n.g. = not given, no = not tested, n.a. not applicable, Headings: Plants: trit. = triticale, Application: Path = pathology, Fung: fungicide research, Res = resistance research, tests, Gen = genetic aspects, QTL: quantitative traits locus Medium to increase fungi: LBA = lima bean medium MBA = mung bean agar, MBM mungo been medium liquid PDA = potato dextrose agar, CMC = carboxyl methyl cellulose, SNA = synthetic nutrient-poor agar, Inoculation of ears: S = spray, P = point, *Fusarium* spp.: gram. = graminearum, culm. = culmorum, Fg + Fc: mixture of the two species. No. of isolates: *v/v* volume/volume, when 4, each have 25% in the pooled inoculum, nFg, nFc, = mixture of the two species without giving the number of the isolates, therefor “n” before, Conidium concentration: 1 × 10^4^, 1 × 10^5^: = two different concentrations were used in the same paper, FHB (Fusarium head blight) Visual: * = 20 days after inoculations, I = Incidence, S = severity, VSS 1–9 = visual scale 1–9, DI = disease index, DAI = Days after inoculation.: AUDPC: area under disease progress curve, FDK = Fusarium damaged kernel, DON: deoxynivalenol.

**Table 2:** Winter wheat genotypes in the tables and figures, Szeged, 2013–2015.

Genotype	Resistance Class
GK Fény	MR
GK Garaboly	S
GK Csillag	MR
GK Futár	S

MR = moderately resistant, S = susceptible, GK is the abbreviation of the Hungarian name of Cereal Research Ltd., as breeding institute.

**Table 3:** Response of the wheat cultivars to the different isolates and their mixtures. Disease index data (%) across experiments, Szeged, 2013–2015.

Isolates			Cultivars, DI%																Mean			Counted			Mean/	
**Table 3A/Mixtures**			**GK Garaboly**					**GK Csillag**					**GK Fény**			**GK Futár**									**Counted%**	
1 Fc 12375			35.83					20.96					22.18			25.26			26.06			26.06			100.00	
2 Fc 52.10			5.42					5.75					4.05			5.09			5.08			5.08			100.00	
3 Fg 19.42			60.94					38.68					47.70			55.78			**50.78** ^1^			50.78			100.00	
4 Fg 13.38			12.30					11.82					10.43			11.42			11.49			11.49			100.00	
1 + 2			26.53					20.17					26.47			25.74			24.73			15.57			158.84	
1 + 3			46.24					31.55					32.24			37.22			**36.81**			38.42			95.83	
1 + 4			25.60					20.46					21.54			24.13			22.93			25.39			90.32	
2 + 3			49.37					36.98					39.10			43.39			**42.21**			27.93			151.16	
2 + 4			6.85					6.55					5.48			5.95			6.21			8.28			74.93	
3 + 4			45.38					32.24					38.52			40.27			**39.10**			31.13			125.60	
1 + 2 + 3			40.62					32.58					31.15			39.04			**35.85**			27.30			131.30	
1 + 2 + 4			27.37					21.95					21.33			22.63			23.32			14.21			164.11	
1 + 3 + 4			34.64					31.04					29.13			34.31			**32.28**			29.44			109.64	
2 + 3 + 4			45.99					35.45					37.09			36.89			**38.86**			22.45			173.09	
1 + 2 + 3 + 4			35.83					32.36					29.15			32.18			**32.38**			23.35			138.66	
Control			0.00					0.00					0.00			0.00			0.00			0.00			0.00	
Mean			31.18					23.66					24.72			27.45			26.75			22.30			128.49 ^2^	
LSD 5% mix.																			2.06							
LSD 5% between cultivars (cvs)																			1.06							
LSD 5% between any data in the table (genotype x inoculum, AxC interaction): 4.12																										
^1^ Bold printed in Mean: Isolate 3 and its mixtures, ^2^ Mean for mixtures only																										
1, 2, 3, 4 = Isolates to be mixed per se. 1 + 2 = mix Is. 1 and 2, 1 + 2 + 3 = Is. 1 and 2 and 3, etc.																										
**Table 3B/Correlations**						**GK Garaboly**			**GK Csillag**							**GK Fény**						**GK Futár**				
GK Csillag						0.9676 ***																				
GK Fény						0.9825 ***			0.9730 ***																	
GK Futár						0.9854 ***			0.9735 ***							0.9898 ***										
*** *p* = 0.001																										
**Table 3C/Inocula**	**3**	**2 + 3**		**3 + 4**	**2 + 3 + 4**		**1 + 3**				**1 + 2 + 3**	**1 + 2 + 3 + 4**		**1 + 3 + 4**			**1**			**1 + 2**	**1 + 2 + 4**		**1 + 4**	**4**		**2 + 4**
2 + 3	0.95																									
3 + 4	0.98 *	0.94																								
2 + 3 + 4	0.78	0.91		0.85																						
1 + 3	0.89	0.99 *		0.89	0.95																					
1 + 2 + 3	0.87	0.91		0.77	0.70		0.89																			
1 + 2 + 3 + 4	0.55	0.77		0.53	0.78		0.85				0.81															
1 + 3 + 4	0.76	0.82		0.64	0.59		0.82				0.98 *	0.82														
1	0.86	0.97 *		0.89	0.98 *		0.99 *				0.83	0.84		0.74												
1 + 2	0.82	0.65		0.86	0.53		0.54				0.43	0.02		0.27			0.54									
1 + 2 + 4	0.75	0.92		0.79	0.97 *		0.97 *				0.80	0.91		0.74			0.98 *			0.37						
1 + 4	0.98 *	0.98 *		0.94	0.81		0.94				0.94	0.70		0.86			0.90			0.69	0.83					
4	0.31	0.56		0.26	0.58		0.67				0.68	0.96 *		0.74			0.65			−0.27	0.76		0.49			
2 + 4	0.18	0.47		0.18	0.59		0.60				0.52	0.92		0.58			0.61			−0.35	0.74		0.36	0.97 *		
2	−0.07	0.18		−0.16	0.18		0.29				0.42	0.74		0.55			0.26			−0.63	0.41		0.13	0.90		0.88
* significant at *p* = 0.05, limit r = 0.95																										
1, 2, 3, 4 = Isolates to be mixed per se. 1 + 2 = mix Is. 1 and 2, 1 + 2 + 3 = Is. 1 and 2 and 3, etc.																										

**Table 4:** Response during the experiments to the different isolates and their mixtures. Disease index data (%) across cultivars, Szeged, 2013–2015.

**Table 4A/Isolates**								**Years**											**Mean**	
**Mixtures**					**2013**			**2014**			**2015a**				**2015b**					
1 Fc 12375					40.13			9.17			26.93				28.00				26.06	
2 Fc 52.10					8.33			1.09			4.88				6.01				5.08	
3 Fg 19.42					61.13			26.00			54.00				61.98				50.78	
4 Fg 13.38					14.03			6.62			11.78				13.54				11.49	
1 + 2					44.96			5.07			23.29				25.58				24.73	
1 + 3					66.19			15.50			31.58				33.98				36.81	
1 + 4					48.21			5.73			18.58				19.21				22.93	
2 + 3					70.94			5.33			44.04				48.54				42.21	
2 + 4					10.75			4.10			4.62				5.36				6.21	
3 + 4					68.00			5.60			43.06				39.75				39.10	
1 + 2 + 3					57.63			17.44			32.31				36.02				35.85	
1 + 2 + 4					52.99			1.85			17.63				20.81				23.32	
1 + 3 + 4					56.95			5.09			30.25				36.83				32.28	
2 + 3 + 4					53.24			8.97			44.56				48.65				38.86	
1 + 2 + 3 + 4					54.06			11.14			31.54				32.77				32.38	
Mean					44.22			8.04			26.19				28.57				26.75	
LSD 5%					4.12			4.12			4.12				4.12				2.06	
1, 2, 3, 4 = Isolates to be mixed per se. 1 + 2 = mix Is. 1 and 2, 1 + 2 + 3 = Is. 1 and 2 and 3, etc.																				
**Table 4B/Correlations**					**2013**			**2014**				**2015a**					**2015b**			
2014					0.5036															
2015a					0.8734 ***			0.6682 **												
2015b					0.8577 ***			0.6952 **				0.9903 ***								
*** *p* = 0.001, ** *p* = 0.01.																				
**Table 4C/Inocula**	**1**	**2**	**3**	**4**	**1 + 2**	**1 + 3**	**1 + 4**		**2 + 3**	**2 + 4**			**3 + 4**	**1 + 2 + 3**		**1 + 2 + 4**		**1 + 3 + 4**		**2 + 3 + 4**
2	0.99 *																			
3	0.91	0.93																		
4	0.94	0.96 *	0.99 *																	
1 + 2	0.99 *	0.98 *	0.85	0.89																
1 + 3	0.94	0.93	0.73	0.79	0.98 *															
1 + 4	0.92	0.91	0.69	0.76	0.97 *	1.00 *														
2 + 3	1.00 *	1.00 *	0.93	0.96 *	0.98 *	0.92	0.90													
2 + 4	0.82	0.82	0.54	0.63	0.90	0.97 *	0.97 *		0.80											
3 + 4	1.00 *	0.98 *	0.88	0.92	0.99 *	0.94	0.93		0.99 *	0.83										
1 + 2 + 3	0.97 *	0.96 *	0.80	0.86	0.99 *	0.99 *	0.99 *		0.96 *	0.94			0.97 *							
1 + 2 + 4	0.94	0.93	0.73	0.79	0.98 *	1.00 *	1.00 *		0.92	0.97 *			0.94	0.99 *						
1 + 3 + 4	0.99 *	1.00 *	0.90	0.94	0.99 *	0.96 *	0.94		0.99 *	0.86			0.98 *	0.98 *		0.96 *				
2 + 3 + 4	0.94	0.95 *	0.99 *	0.99 *	0.89	0.78	0.75		0.96 *	0.60			0.93	0.84		0.78		0.92		
1 + 2 + 3 + 4	0.99 *	0.98 *	0.85	0.89	1.00 *	0.98 *	0.97 *		0.98 *	0.89			0.99 *	0.99 *		0.98 *		0.99 *		0.89
* *p* = 5%, Limit: r = 0.95.																				
1,2,3,4 = Isolates to be mixed per se. 1 + 2 = mix Is. 1 and 2, 1 + 2 + 3 = Is. 1 and 2 and 3, etc.																				

**Table 5:** Response of the wheat cultivars to the different isolates and their mixtures. FDK data (%) across experiments, Szeged, 2013–2015.

**Table 5A/Isolates**		**Cultivars**														**Mean**				**Counted**			**Mean**	
**Mixtures**		**GK Garaboly**					**GK Futár**		**GK Fény**					**GK Csillag**						**Counted**			**Counted%**	
1 Fc 12375		56.88					43.44		36.69					38.06		43.77				43.77			100.00	
2 Fc 52.10		9.39					5.51		6.38					5.05		6.58				6.58			100.00	
3 Fg 19.42		82.63					73.00		68.75					53.13		**69.38** ^1^				69.38			100.00	
4 Fg 13.38		26.56					22.50		17.50					20.44		21.75				21.75			100.00	
1 + 2		46.25					47.63		44.38					32.33		42.64				25.17			169.41	
1 + 3		68.94					54.19		45.31					37.50		**51.48**				56.57			91.01	
1 + 4		48.44					42.44		36.69					31.63		39.80				43.20			92.11	
2 + 3		70.25					54.56		43.89					48.82		**54.38**				37.98			143.19	
2 + 4		18.88					11.94		11.81					11.88		13.63				14.16			96.19	
3 + 4		66.13					61.81		59.88					41.88		**57.42**				45.56			126.03	
1 + 2 + 3		79.38					64.38		56.56					48.44		**62.19**				39.91			155.83	
1 + 2 + 4		50.75					36.51		34.08					34.71		39.01				24.03			162.34	
1 + 3 + 4		63.31					51.13		46.44					49.75		**52.66**				44.96			117.11	
2 + 3 + 4		72.13					59.81		51.91					56.06		**59.98**				32.57			184.16	
1 + 2 + 3 + 4		70.94					53.75		53.13					57.19		**58.75**				35.37			166.11	
Mean		55.39					45.51		40.89					37.79		44.89				33.81			136.68 ^2^	
LSD 5% Mix.		7.65					7.65		7.65					7.65		7.65				3.82				
LSD 5% cv																				1.97				
^1^ Bold printed in Mean: Isolate 3 and its mixtures, ^2^ Mean only for mixtures,																								
1, 2, 3, 4 = Isolates to be mixed per se. 1 + 2 = mix Is. 1 and 2, 1 + 2 + 3 = Is. 1 and 2 and 3, etc.																								
**Table 5B/Correlations**					**GK Garaboly**							**GK Futár**						**GK Fény**						
GK Futár					0.9735 ***																			
GK Fény					0.9492 ***							0.9866 ***												
GK Csillag					0.9521 ***							0.9169 ***						0.9041 ***						
*** *p* = 0.001.																								
**Table 5C/inocula**	**1**		**2**	**3**		**4**		**1 + 2**		**1 + 3**	**1 + 4**		**2 + 3**		**2 + 4**		**3 + 4**		**1 + 2 + 3**		**1 + 2 + 4**	**1 + 3 + 4**		**2 + 3 + 4**
2	0.88																							
3	0.79		0.81																					
4	0.95 *		0.70	0.64																				
1 + 2	0.48		0.49	0.90		0.34																		
1 + 3	0.94		0.87	0.95 *		0.85		0.74																
1 + 4	0.90		0.81	0.97 *		0.81		0.81		0.99 *														
2 + 3	0.99 *		0.82	0.73		0.98 *		0.40		0.91	0.87													
24	0.95 *		0.96 *	0.72		0.85		0.35		0.87	0.80		0.93											
3 + 4	0.62		0.69	0.97 *		0.45		0.97 *		0.85	0.89		0.54		0.54									
1 + 2 + 3	0.94		0.88	0.95 *		0.84		0.74		1.00 *	0.99 *		0.90		0.87		0.85							
1 + 2 + 4	0.98 *		0.94	0.75		0.90		0.40		0.90	0.85		0.97 *		0.99 *		0.58		0.91					
1 + 3 + 4	0.99 *		0.86	0.69		0.95 *		0.32		0.87	0.82		0.99		0.97 *		0.49		0.87		0.99 *			
2 + 3 + 4	0.99 *		0.82	0.71		0.98 *		0.38		0.90	0.85		1.00 *		0.93		0.52		0.89		0.97 *	0.99 *		
1 + 2 + 3 + 4	0.91		0.89	0.56		0.85		0.15		0.76	0.68		0.91		0.98 *		0.35		0.77		0.96 *	0.96 *		0.92
* *p* = 5%, Limit: r = 0.95.																								
1, 2, 3, 4 = Isolates to be mixed per se. 1 + 2 = mix Is. 1 and 2, 1 + 2 + 3 = Is. 1 and 2 and 3, etc.																								

**Table 6:** Response of wheat to the different isolates and their mixtures. FDK data (%) of the experiments across wheat cultivars, Szeged, 2013–2015.

Isolates				Experiments											Mean	
**Table 6A/Mixtures**				**2013**		**2014**				**2015a**	**2015b**					
1 Fc 12375				69.69		14.75				44.06	46.56				43.77	
2 Fc 52.10				4.22		0.73				11.31	10.06				6.58	
3 Fg 19.42				84.25		39.88				74.06	79.31				69.38	
4 Fg 13.38				39.06		7.50				19.69	20.75				21.75	
1 + 2				65.94		5.58				46.56	52.50				42.64	
1 + 3				79.25		21.69				55.94	49.06				51.48	
1 + 4				70.00		9.81				39.06	40.31				39.80	
2 + 3				82.38		6.14				61.25	67.75				54.38	
2 + 4				23.94		5.13				13.13	12.31				13.63	
3 + 4				84.44		9.94				70.94	64.38				57.42	
1 + 2 + 3				75.00		35.00				72.50	66.25				62.19	
1 + 2 + 4				72.40		0.84				38.13	44.69				39.01	
1 + 3 + 4				78.63		6.38				61.88	63.75				52.66	
2 + 3 + 4				82.50		15.59				69.63	72.19				59.98	
1 + 2 + 3 + 4				82.50		21.56				68.44	62.50				58.75	
Mean				66.28		13.37				49.77	50.16				44.89	
LSD 5%				7.65		7.65				7.65	7.65				3.82	
LSD Experiment 5%															1.97	
1, 2, 3, 4 = Isolates to be mixed per se. 1 + 2 = mix Is. 1 and 2, 1 + 2 + 3 = Is. 1 and 2 and 3, etc.																
**Table 6B/Correlations**				**2013**					**2014**					**2015a**		
2014				0.5344 *												
2015a				0.9278 ***					0.6786 **							
2015b				0.9392 ***					0.6262 **					0.9813 ***		
*** *p* = 0.001, ** *p* = 0.01, * *p* = 0.05.																
**Table 6C/Inocula**	**1**	**2**	**3**	**4**	**1 + 2**	**1 + 3**	**1 + 4**	**2 + 3**		**2 + 4**	**3 + 4**	**1 + 2 + 3**	**1 + 2 + 4**		**1 + 3 + 4**	**2 + 3 + 4**
2	0.36															
3	0.94	0.65														
4	0.98 *	0.15	0.84													
1 + 2	0.97 *	0.55	0.99 *	0.90												
1 + 3	0.99 *	0.34	0.90	0.97 *	0.94											
1 + 4	1.00 *	0.28	0.90	0.99 *	0.95 *	0.99 *										
2 + 3	0.96 *	0.59	1.00 *	0.88	1.00 *	0.93	0.93									
2 + 4	0.97	0.16	0.83	1.00 *	0.89	0.98 *	0.99 *	0.87								
3 + 4	0.95 *	0.62	0.98 *	0.87	0.98 *	0.95 *	0.93	0.99 *		0.87						
1 + 2 + 3	0.91	0.69	0.97 *	0.81	0.96 *	0.91	0.88	0.97 *		0.82	0.99 *					
1 + 2 + 4	1.00 *	0.36	0.94	0.97 *	0.98 *	0.98 *	0.99 *	0.97 *		0.96 *	0.95 *	0.90				
1 + 3 + 4	0.96 *	0.62	0.99 *	0.87	1.00 *	0.94	0.93	1.00 *		0.87	0.99 *	0.98 *	0.96 *			
2 + 3 + 4	0.94	0.66	1.00 *	0.84	0.99 *	0.92	0.91	1.00 *		0.84	0.99 *	0.98 *	0.94		1.00 *	
1 + 2 + 3 + 4	0.97 *	0.56	0.97 *	0.90	0.98 *	0.97 *	0.95 *	0.98 *		0.90	1.00 *	0.99 *	0.96 *		0.99 *	0.98 *
* *p* = 5%. Limit: r = 0.95.																
1,2,3,4 = Isolates to be mixed per se. 1 + 2 = mix Is. 1 and 2, 1 + 2 + 3 = Is. 1 and 2 and 3, etc.																

**Table 7:** Response of the wheat cultivars to the different isolates and their mixtures. DON data (mg/kg) across experiments, Szeged, 2013–2015.

Isolates			Cultivars, DON, mg/kg															Mean		Counted				Mean */	
**Table 7A /Mixtures**			**GK Garaboly**				**GK Futár**				**GK Fény**			**GK Csillag**				**Mean**						**Counted%**	
1 Fc 12375			70.42				48.28				36.84			35.57				47.78		47.78				100.00	
2 Fc 52.10			18.99				7.95				10.34			7.84				11.28		11.28				100.00	
3 Fg 19.42			158.56				117.39				94.60			53.95				**106.13** ^1^		106.13				100.00	
4 Fg 13.38			3.78				4.40				4.26			3.67				4.03		4.03				100.00	
1 + 2			58.56				63.62				41.00			28.50				47.92		29.53				162.28	
1 + 3			126.33				66.39				33.17			31.40				**64.32**		76.95				83.59	
1 + 4			45.66				33.92				28.79			20.02				32.10		25.90				123.91	
2 + 3			116.63				66.29				51.69			60.90				**73.88**		58.70				125.85	
2 + 4			10.29				4.50				5.39			5.43				6.40		7.66				83.61	
3 + 4			124.46				70.36				78.36			40.47				**78.41**		55.08				142.37	
1 + 2 + 3			120.70				85.78				44.61			38.35				**72.36**		55.06				131.42	
1 + 2 + 4			61.18				29.92				26.34			27.53				36.24		28.58				126.82	
1 + 3 + 4			97.25				64.18				41.66			54.87				**64.49**		52.64				122.50	
2 + 3 + 4			125.21				89.21				54.30			68.41				**84.28**		40.48				208.21	
1 + 2 + 3 + 4			106.08				59.19				56.08			58.50				**69.96**		42.30				165.38	
Control			3.38				2.39				1.60			2.51				2.47		2.47				100.00	
Mean			77.97				50.86				38.07			33.62				50.13		40.28				134.18 ^2^	
LSD 5%			18.71				18.71				18.71			18.71				9.35							
LSD 5% var.																		4.67							
^1^ Bold printed: Isolate 3 and its combinations, ^2^ Mean only for mixtures.																									
1,2,3,4 = Isolates to be mixed per se. 1 + 2 = mix Is. 1 and 2, 1 + 2 + 3 = Is. 1 and 2 and 3, etc.																									
**Table 7B/Correlations**					**GK Garaboly**				**GK Futár**								**GK Fény**				**GK Csillag**				
GK Futár					0.9516 ***																				
GK Fény					0.8997 ***				0.9106 ***																
GK Csillag					0.8740 ***				0.8467 ***								0.8097 ***								
Mean					0.9846 ***				0.9760 ***								0.9434 ***				0.9095 ***				
*** *p* = 0.001.																									
**Table 7C/Inocula**	**Control**	**4**		**2 + 4**		**2**		**1 + 4**		**1 + 2 + 4**		**1**	**1 + 2**		**1 + 3**	**1 + 3 + 4**			**1 + 2 + 3 + 4**	**1 + 2 + 3**		**2 + 3**	**3 + 4**		**2 + 3 + 4**
4	−0.60																								
2 + 4	0.79	−0.55																							
2	0.69	−0.38		0.98 *																					
1 + 4	0.63	0.08		0.76		0.85																			
1 + 2 + 4	0.86	−0.43		0.97 *		0.96 *		0.87																	
1	0.84	−0.21		0.87		0.88		0.95 *		0.96 *															
1 + 2	0.39	0.49		0.29		0.40		0.83		0.50		0.72													
1 + 3	0.85	−0.21		0.86		0.88		0.94		0.96 *		1.00 *	0.72												
1 + 3 + 4	0.95 *	−0.40		0.86		0.82		0.83		0.95 *		0.97 *	0.60		0.97 *										
1 + 2 + 3 + 4	0.86	−0.47		0.98 *		0.96 *		0.84		1.00 *		0.95 *	0.46		0.94	0.94									
1 + 2 + 3	0.78	−0.03		0.74		0.77		0.96 *		0.88		0.98 *	0.84		0.98 *	0.93			0.85						
2 + 3	0.92	−0.45		0.94		0.91		0.85		0.99 *		0.97 *	0.52		0.97 *	0.98 *			0.99 *	0.90					
3 + 4	0.54	−0.02		0.84		0.93		0.96 *		0.88		0.89	0.65		0.88	0.76			0.87	0.85		0.83			
2 + 3 + 4	0.93	−0.29		0.81		0.78		0.86		0.92		0.97 *	0.69		0.98 *	0.99 *			0.91	0.96 *		0.96 *	0.76		
3	0.58	0.15		0.71		0.81		1.00 *		0.82		0.92	0.86		0.92	0.80			0.80	0.95 *		0.81	0.94		0.84
* *p* = 0.05. Limit: r = 0.95																									
1,2,3,4 = Isolates to be mixed per se. 1 + 2 = mix Is. 1 and 2, 1 + 2 + 3 = Is. 1 and 2 and 3, etc.																									

**Table 8:** Response of wheat to the different isolates and their mixtures. DON data (mg/kg) of the experiments across wheat cultivars, Szeged, 2013–2015.

Isolates					Experiments																	Mean	
**Table 8A/Mixtures**					**2013**			**2014**					**2015a**				**2015b**						
1 Fc 12375					32.98			5.74					72.85				79.55					47.78	
2 Fc 52.10					1.38			0.70					20.65				22.39					11.28	
3 Fg 19.42					52.36			20.82					166.73				184.60					106.13	
4 Fg 13.38					1.86			1.05					6.59				6.63					4.03	
1 + 2					24.39			3.03					76.96				87.29					47.92	
1 + 3					46.62			9.37					111.46				89.86					64.32	
1 + 4					29.58			1.59					50.65				46.58					32.10	
2 + 3					49.85			2.44					102.20				141.03					73.88	
2 + 4					2.81			1.75					11.59				9.46					6.40	
3 + 4					51.53			4.48					142.16				115.50					78.41	
1 + 2 + 3					35.26			15.14					127.27				111.77					72.36	
1 + 2 + 4					29.98			0.61					58.86				55.51					36.24	
1 + 3 + 4					36.11			3.41					115.20				103.24					64.49	
2 + 3 + 4					52.11			5.91					132.52				146.59					84.28	
1 + 2 + 3 + 4					43.16			7.91					129.64				99.14					69.96	
Control					1.51			1.12					3.98				3.27					2.47	
Mean Ecp.					30.72			5.32					83.08				81.40					50.13	
LSD 5%					18.71			18.71					18.71				18.71					4.67	
1, 2, 3, 4 = Isolates to be mixed per se. 1 + 2 = mix Is. 1 and 2, 1 + 2+3 = Is. 1 and 2 and 3, etc.																							
**Table 8B/Correlations**					**GK Garaboly**						**GK Futár**					**GK Fény**				**GK Csillag**			
**B/Correlations**					**2013**						**2014**					**2015a**				**2015b**			
2014					0.5552 *																		
2015a					0.9333 ***						0.7251 **												
2015b					0.9171 ***						0.6934 **					0.9458 ***							
Mean					0.9490 ***						0.7217 **					0.9870 ***				0.9836 ***			
*** *p* = 0.001, ** *p* = 0.01, * *p* = 0.05.																							
**Table 8C/Inocula**	**1**	**2**	**3**	**4**		**1 + 2**	**1 + 3**		**1 + 4**	**2 + 3**		**2 + 4**		**3 + 4**	**1 + 2 + 3**			**1 + 2 + 4**	**1 + 3 + 4**		**2 + 3 + 4**		**1 + 2 + 3 + 4**
2	0.95 *																						
3	0.99 *	0.99 *																					
4	0.97 *	0.99 *	1.00 *																				
1 + 2	0.99 *	0.98 *	1.00 *	0.99 *																			
1 + 3	0.96 *	0.92	0.94	0.95 *		0.95 *																	
1 + 4	0.97 *	0.86	0.92	0.91		0.93	0.97																
2 + 3	0.98 *	0.93	0.97 *	0.94		0.98 *	0.90		0.92														
2 + 4	0.94	0.97 *	0.96 *	0.98 *		0.95 *	0.97 *		0.90	0.87													
3 + 4	0.97 *	0.93	0.95 *	0.96 *		0.96 *	1.00 *		0.97 *	0.90		0.97 *											
1 + 2 + 3	0.97 *	0.98 *	0.98 *	0.99 *		0.97 *	0.98 *		0.92	0.91		1.00 *		0.98 *									
1 + 2 + 4	0.98 *	0.90	0.95 *	0.94 *		0.96 *	0.98 *		1.00	0.94		0.93		0.98 *	0.95 *								
1 + 3 + 4	0.98 *	0.96 *	0.98 *	0.99 *		0.98 *	0.99 *		0.96 *	0.93		0.98 *		0.99 *	0.99 *			0.98 *					
2 + 3 + 4	1.00 *	0.97 *	0.99 *	0.98 *		1.00 *	0.96 *		0.96 *	0.98 *		0.95 *		0.97 *	0.97 *			0.98 *	0.98 *				
1 + 2 + 3 + 4	0.95 *	0.93	0.94	0.96 *		0.94	1.00 *		0.95 *	0.88		0.98 *		1.00 *	0.99 *			0.97 *	0.99 *		0.95 *		
Control	0.94	0.96 *	0.95 *	0.98*		0.95 *	0.98 *		0.91	0.87		1.00 *		0.98 *	0.99 *			0.93	0.98 *		0.94		0.99 *
* *p* = 0.05. Limit: r = 0.95.																							
1, 2, 3, 4 = Isolates to be mixed per se. 1 + 2 = mix Is. 1 and 2, 1 + 2+3 = Is. 1 and 2 and 3, etc.																							

**Table 9:** Response of wheat to the different isolates and their mixtures. ANOVAs for the three traits, FHB, FDK and DON, Szeged, 2013–2015.

**Table 9A/Source of**		**DI ^a^**				**FDK ^b^**					**DON ^c^**			
**Variance**	**df**	**MS**	**F**		***p***	**MS**	**F**	***p***	**df**		**MS**		**F**	***p***
Variety A	3	3076.8	86.8		***	14,160.0	116.2	***	3		44,671.8		61.3	***
Experiment B	3	59,962.0	1690.8		***	120,215.8	986.8	***	3		322,433.4		442.3	***
Inoculum C	14	11,287.9	318.3		***	21,562.8	177.0	***	15		53,877.0		73.9	***
A × B	9	110.6	3.1		***	919.7	7.5	***	9		33,981.2		46.6	***
A × C	42	156.4	4.4		***	387.8	3.2	***	45		8180.8		11.2	***
B × C	42	1235.1	34.8		***	1699.9	14.0	***	45		14,704.9		20.2	***
A × B × C	126	62.4	1.8		n.s.	203.2	1.7	n.s.	135		621.8		0.9	n.s.
Within	720	35.5				121.89			768		729.0			
Total	959								1023					
*** *p* = 0.001, n.s. = not significant. Analysis between two-way interactions and main effects.															
**Table 9B/Interactions, and df ^d^**				**df 9A**		**F**	***p***	**F**		***p***		**df**		**F**	***p***
A × B, df = 9				A, df 3		27.81	***	15.40		***		A, df 3		1.31	n.s.
				B, df 3		542.15	***	130.71		***		B, df 3		9.49	**
A × C, df 42				A, df 3		19.67	***	36.51		***		A, df 3		5.46	*
				C, df 14		72.17	***	54.80		***		C, df 15		6.59	***
BcC, df 42				B, df3		48.55	***	70.72		***		B × C,45; B, df 3		21.93	***
				C, df 14		9.14	***	12.68		***		B × C,45; B, df 15		2.31	*
*** *p* = 0.001, ** *p* = 0.01, * *p* = 0.05, n.s. = not significant. ^a^ = Disease Index, ^b^ = Fusarium damaged kernel, ^c^ = deoxynivalenol, ^d^ = degree of freedom.															

**Table 10:** Correlations between DI, FDK and DON contamination the four cultivars (*n* = 4) for each isolates and mixtures and their means, Szeged, 2013–2015.

Inocula	Correlations between		
	DI/FDK	DI/DON	FDK/DON
1 Fc 12375	0.9894 *	0.9941 *	0.9947 *
2 Fc 52.10	0.0282	0.1054	0.9950 *
3 Fg 19.42	0.9790 *	0.9842 *	0.9870 *
4 Fg 13.38	0.8732	−0.6943	−0.3513
1 + 2	0.9577 *	0.7515	0.8971
1 + 3	0.9760 *	0.9996 *	0.9698 *
1 + 4	0.9907 *	0.9638	0.9904 *
2 + 3	0.9353	0.9084	0.9819 *
2 + 4	0.7020	0.7011	0.9846 *
3 + 4	0.9492	0.9552 *	0.8570
1 + 2 + 3	0.8668	0.9532	0.9754 *
1 + 2 + 4	0.9973 *	0.9943 *	0.9991 *
1 + 3 + 4	0.7703	0.8500	0.9906 *
2 + 3 + 4	0.9100	0.8746	0.9929 *
1 + 2 + 3 + 4	0.8984	0.8695	0.9804 *
Mean ^a^	0.6170	0.6668	0.9968 *
Average of 15 inocula ^b^	0.8549	0.7474	0.8830

* *p* = 0.05, ^a^ Means for all inocula. ^b^ averages of correlations (from inoculum 1 to mixture 1+2+3+4) across all inocula.

## Abbreviations

ANOVA	analysis of variance
AUDPC	area under the disease progress curve
DF	degree of freedom
DI	Disease index
DON	deoxynivalenol
FHB	Fusarium damaged kernel
GK	before variety names: Hungarian abbreviation for Cereal Research institution
LSD	limit of significant difference
QTL	quantitative trait locus
SPE	solid phase extraction column
